# Supplementary material for: Attention Selectively Reshapes the Geometry of Distributed Semantic Representation
Source: Cereb Cortex. 2017 Jun 7;27(8):4277–91. doi: 10.1093/cercor/bhx138 (PMC6248820; doi:10.1093/cercor/bhx138)
Supplement: Supplementary Data [file supplementarymaterial(revised).pdf]

Supplementary Material

Attention selectively reshapes the geometry of distributed semantic representation

Samuel A. Nastase, Andrew C. Connolly, Nikolaas N. Oosterhof, Yaroslav O. Halchenko, J. Swaroop Guntupalli, Matteo Visconti di Oleggio Castello, Jason Gors, M. Ida Gobbini, James V. Haxby

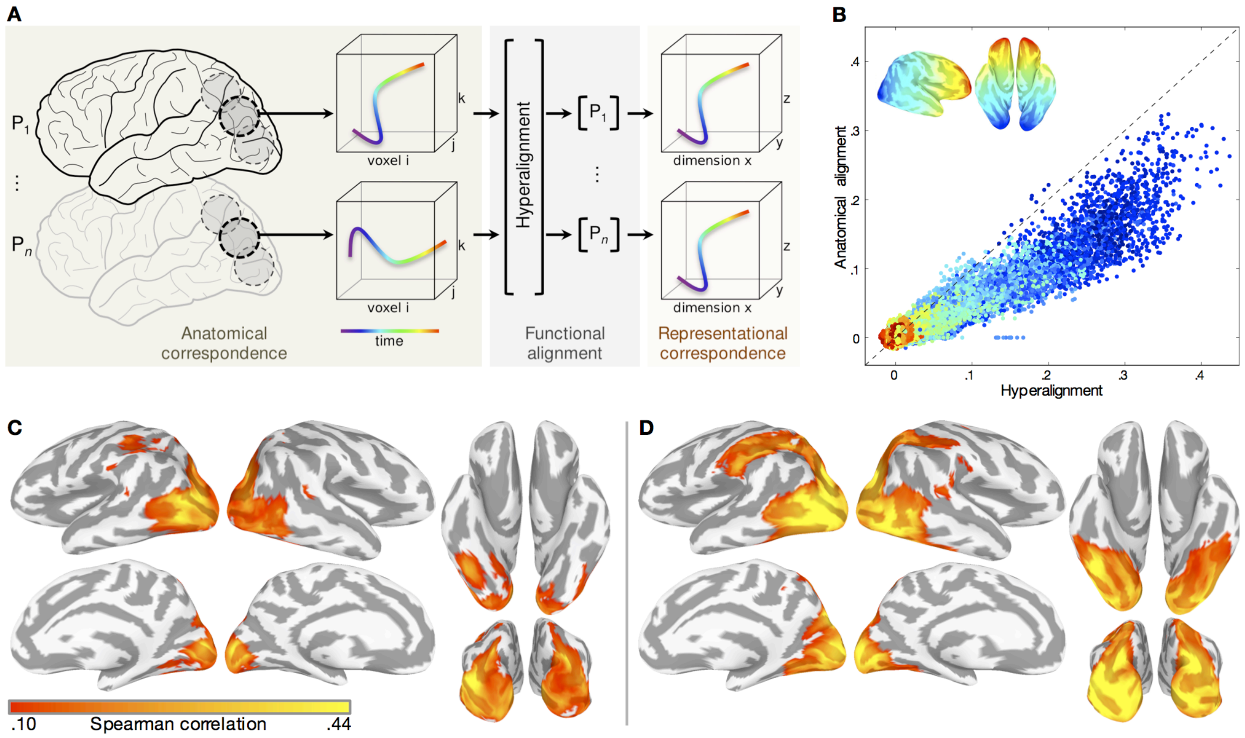

Supplementary Figure 1. Whole-brain searchlight hyperalignment enhances representational correspondence across participants. (*A*) For each surface-based searchlight, the Procrustes transformation is used to rotate each participant's time series of functional response patterns to the *Life* movie stimulus into a common space that maximizes representational correspondence across participants. These patterns are depicted as a trajectory of responses in a three-voxel space over time. (*B*) Each point in the scatterplot represents the average inter-participant Spearman correlation of RDMs for both attention tasks in a single searchlight. For each surface-based searchlight, the vectorized upper triangulars of the observed neural RDMs for both attention tasks were concatenated and pairwise Spearman correlations were computed between all participants. The vertical axis indicates Spearman correlation based on surface-based spherical alignment; the horizontal axis indicates Spearman correlation after surface-based searchlight whole-brain hyperalignment. Deviance from the identity line indicates a strong effect of alignment method on inter-participant similarity of RDMs.

Searchlights are colored according their location on the posterior–anterior axis of the inflated cortical surface. (*C*) Inter-participant Spearman correlation of searchlight RDMs for both attention tasks using anatomical alignment thresholded at .10. (*D*) Average inter-participant Spearman correlation of searchlight RDMs after hyperalignment at the same threshold. Prior to hyperalignment, the maximum mean Spearman correlation was .32 in a searchlight superior to the left lateral occipital sulcus. Following hyperalignment, the maximum mean Spearman correlation was .44 in a searchlight in the left lateral occipital sulcus.

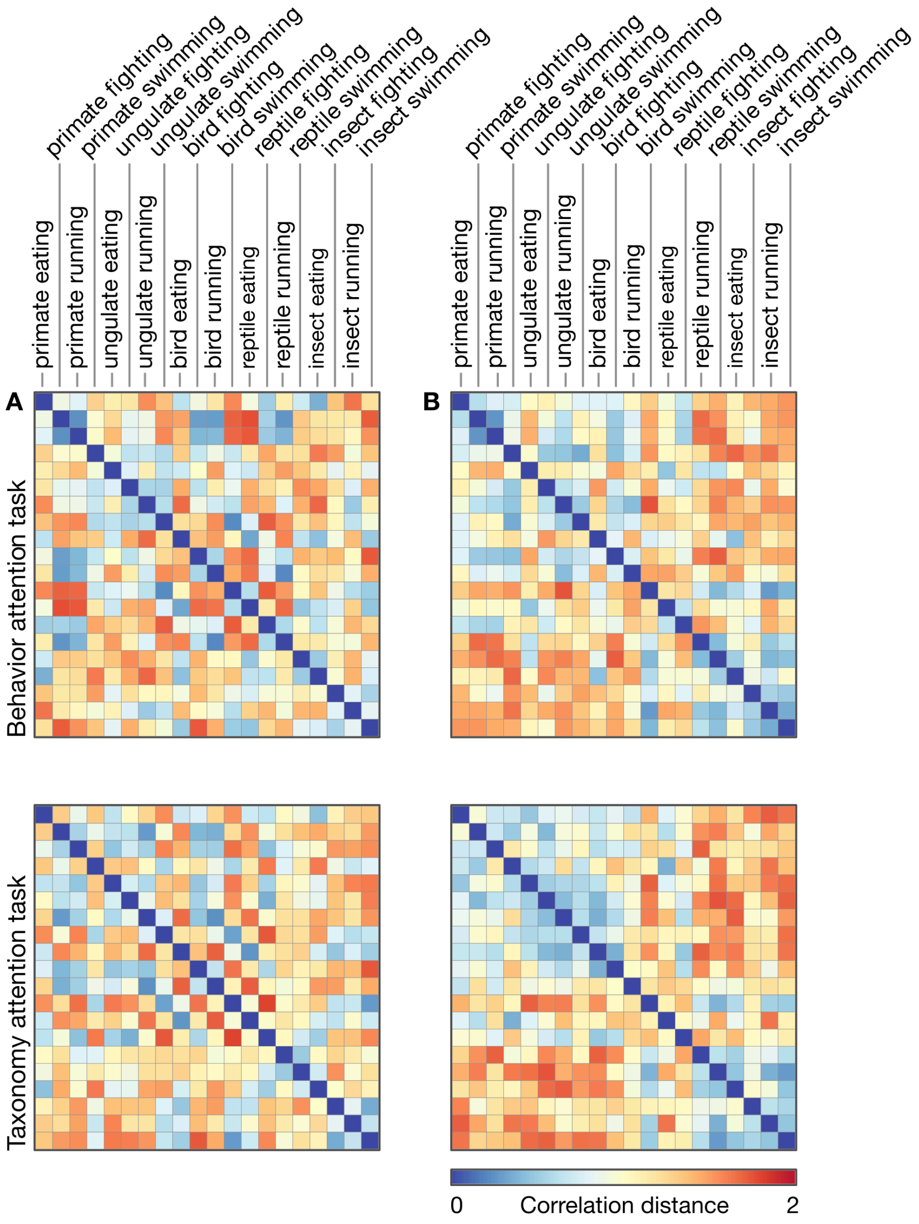

Supplementary Figure 2. Example searchlight representational geometries. The observed neural RDMs for two example searchlights from a representative participant are plotted for the behavior attention task and the taxonomy attention task. (*A*) Observed neural RDM for a searchlight in left lateral occipitotemporal cortex (posterior middle temporal gyrus). Differences in behavioral category representation are reflected in repeated off-diagonal strips. (*B*) Observed neural RDM for a searchlight in left ventral temporal cortex (posterolateral fusiform gyrus).

Taxonomic categories are represented according to an animacy continuum varying systematically in similarity from primates (most animate) to insects (least animate; Connolly et al. 2012; Sha et al. 2015). Example searchlights were not selected to convey an effect of the task manipulation.

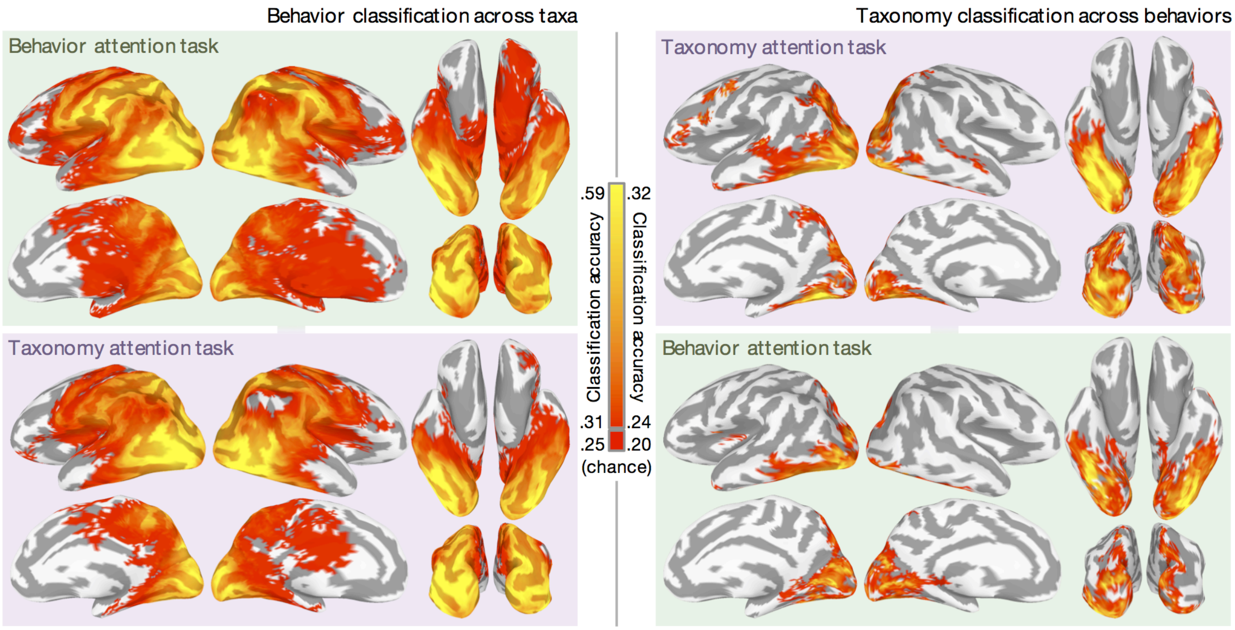

Supplementary Figure 3. Effect of attention on searchlight classification of behavior and taxonomy. Cross-validation was implemented in the following leave-one-category-out fashion: classifiers discriminating the four behaviors (left) were trained on four of the five taxa, and tested on the left-out taxon; classifiers discriminating the five animal taxa (right) were trained on three of the four behaviors and tested on the left-out behavior. This procedure ensured that any information about animal behavior generalizes across animal taxa, and vice versa. Furthermore, classifiers in this cross-validation scheme are always tested on exemplar clips not in the training set, ensuring that classification accuracy is not based solely on low-level visual properties idiosyncratic to particular stimuli. Prior to classification, the GLM was computed separately for each run, yielding 20 beta parameters per run. The maps are qualitatively similar to the representational similarity regression maps reported in Fig. 2, with an average correlation of .83 across conditions prior to thresholding. Chance accuracy for four-

class behavior classification is .25 and chance accuracy for five-class taxonomy classification is .20. Accuracies less than 0.31 for behavior classification and less than .24 for taxonomy classification are plotted as red. Maps are thresholded at $p < .05$ using TFCE, based on a null distribution of searchlight maps generated by permuting the labels of interest within each run and within each category of the crossed factor.

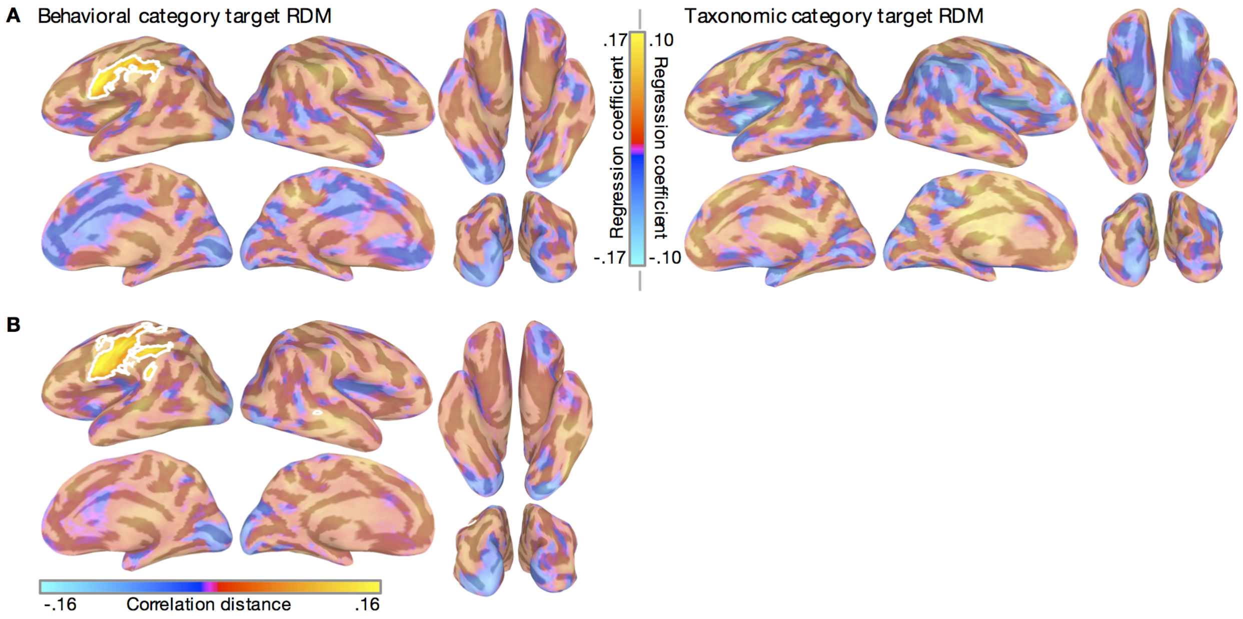

Supplementary Figure 4. Task differences in searchlight representational geometry. (*A*) Attention-related differences in standardized rank regression coefficients were computed for both the behavioral category and taxonomic category target RDMs. Warm colors represent attentional enhancement for the corresponding semantic information. The range of values on the color bar reflects the mean difference in the regression coefficient. (*B*) Cells of the searchlight RDMs capturing within-category distances for both animal behavior and taxonomy were isolated (see Fig. 4) and tested for attentional enhancement of correlation distance. The absolute values of the within-behavior and within-taxon distances were averaged for each searchlight to compute an index of overall task difference in within-category correlation distances. Clusters surviving TFCE-based correction for multiple comparisons at p = .05 (two-tailed test) are displayed at full opacity and outlined with a white contour, while searchlights not surviving TFCE are displayed as partially transparent. TFCE maps were estimated using a

Monte Carlo simulation randomly flipping the attention task label. Note that the trend towards an effect of attention to taxonomy in VT cortex on correlation with the taxonomic RDM was not significant in this searchlight analysis but was strongly significant in the ROI analysis that used larger regions. Searchlights in this case included only 100 voxels and cannot capture the more distributed effects observed in the ROI analysis. Furthermore, searchlight analyses are subjected to conservative multiple comparisons correction because of the large number of searchlights.

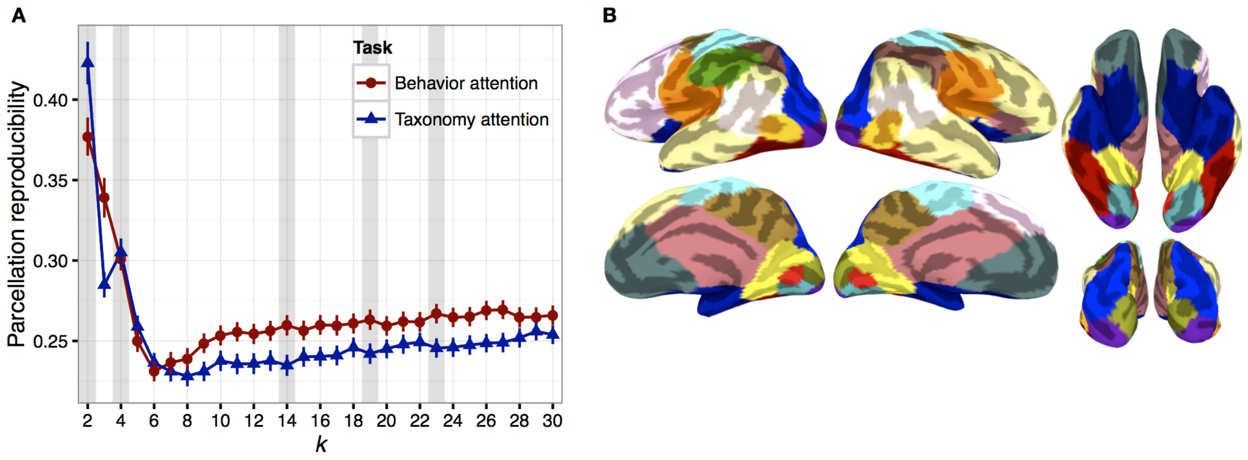

Supplementary Figure 5. Functional parcellation of the cerebral cortex based on representational geometry. (*A*) Parcellation reproducibility was evaluated using split-half resampling across participants (100 partitions per *k*) separately for each attention task. The mean AMI across the 100 partitions is plotted across the values of *k*, with error bars indicating the standard error of the mean across partitions. Vertical gray bars indicate several local maxima spanning the range of *k* tested. Parcellations at these reproducible values of *k* are visualized on the cortical surface in Supplementary Fig. 5. (*B*) Full parcellation at *k* = 19 for the behavior attention task data. Ten parcels from this solution corresponding to the dorsal and ventral visual pathways were further interrogated in the ROI analysis.

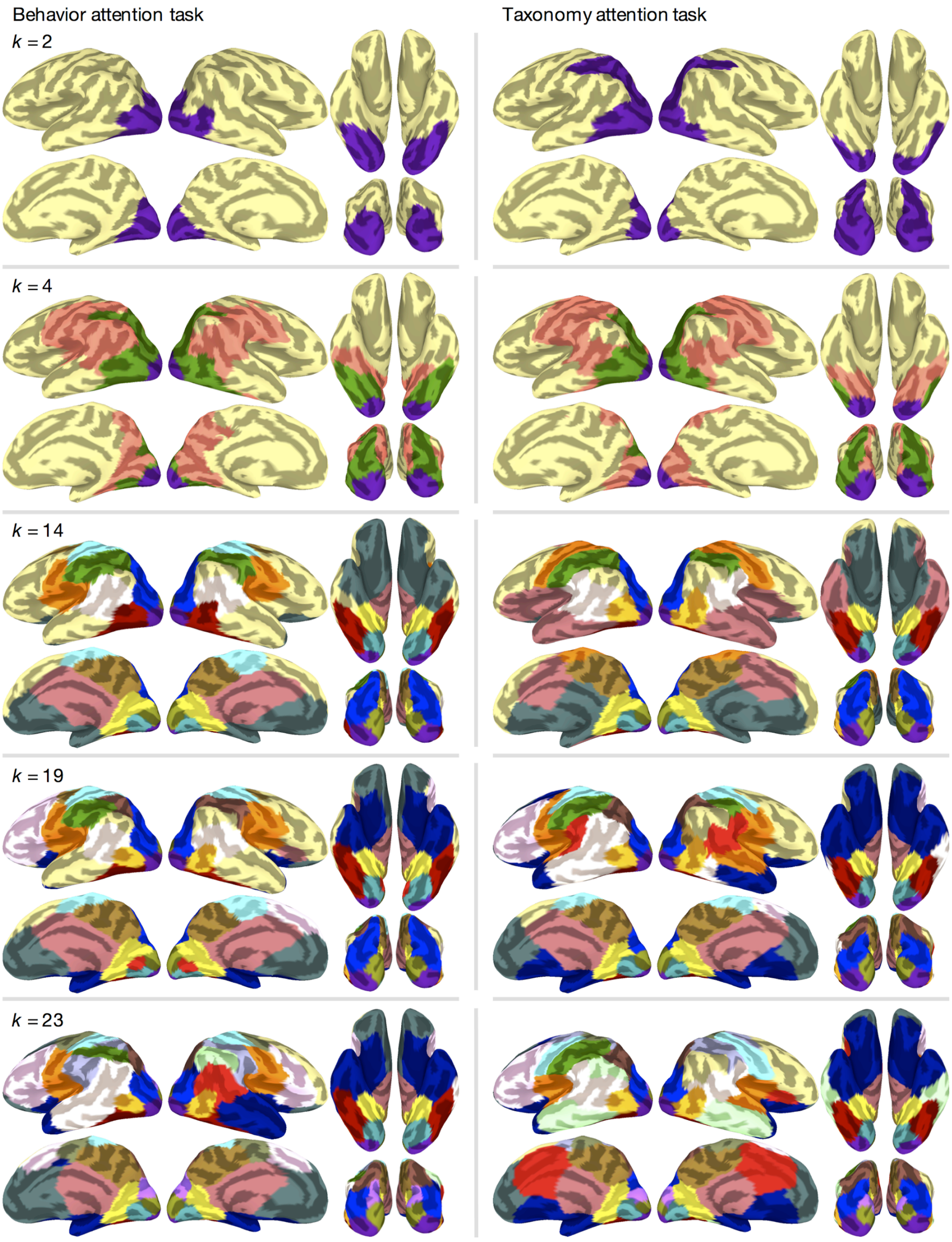

Behavior attention task
Taxonomy attention task
k = 2
k = 4
k = 14
k = 19
k = 23

Supplementary Figure 6. Functional parcellations at reproducible values of *k* for both attention tasks. Parcellation reproducibility was assessed using split-half resampling across participants, and parcellations are depicted for local maxima in parcellation reproducibility (*k* = 2, 4, 14, 19, and 23; corresponding to vertical gray bars in Supplementary Fig. 4*A*). The left column depicts parcellations based on searchlight representational geometries from the behavior attention task and the right column depicts parcellations based on searchlight representational geometries from the taxonomy attention task. The parcellation for the behavior attention task data (left) at *k* = 19 was used for subsequent ROI analysis and is reproduced in Fig. 3 and Supplementary Fig. 4*B*. Colors were assigned manually to avoid similar colors at anatomically adjacent parcels, and to emphasize similar parcels across tasks and values of *k*.

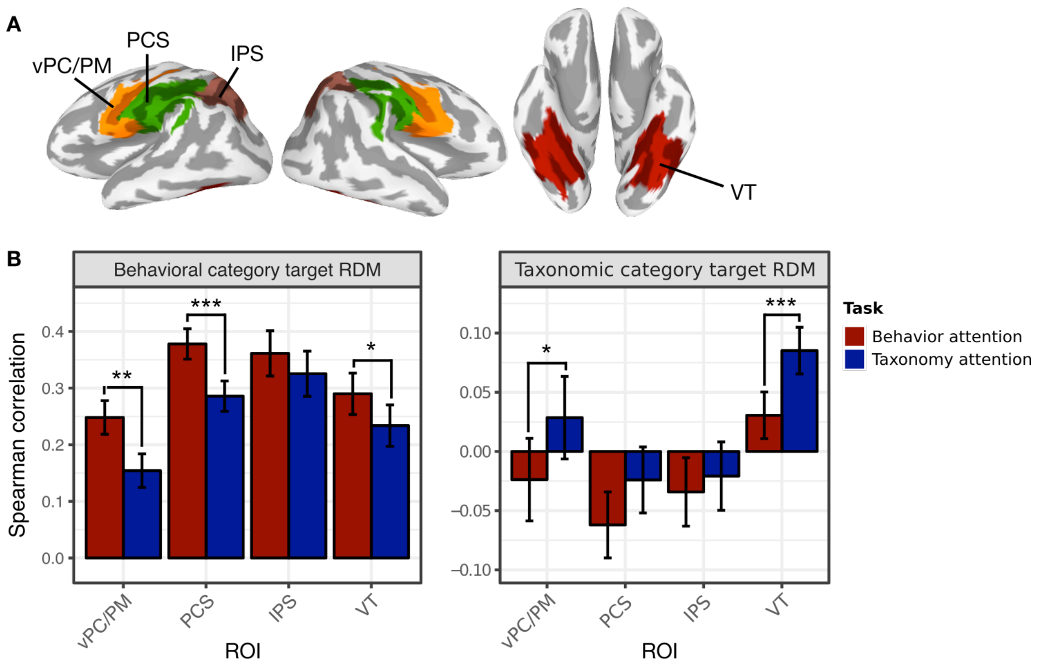

Supplementary Figure 7. Attention alters representational geometry in anatomically-defined ROIs. (*A*) The following anatomically-defined analogues of four key ROIs were extracted from the FreeSurfer cortical surface parcellation (Destrieux et al. 2010). VT: bilateral fusiform gyri (lateral occipitotemporal gyri), collateral sulci (medial occipitotemporal sulci) and lingual sulci, and lateral occipitotemporal sulci; IPS: bilateral intraparietal sulci, transverse parietal sulci, and superior parietal lobules; PCS: bilateral postcentral gyri, postcentral sulci, and supramarginal gyri extending superiorly to $z = 50$; vPC/PM: bilateral precentral gyri, central sulci, and subcentral gyri (central opercula) extending superiorly to $z = 50$. (*B*) Attending to animal behavior increased Spearman correlations between the observed neural RDM and the behavioral category target RDM in vPC/PM ($p = .001$), PCS ($p < .001$), and VT ($p = .030$). Attending to animal taxonomy increased correlations between the observed neural RDM and the taxonomic category target RDM in vPC/PM ($p = .043$) and VT ($p < .001$). Error bars indicate bootstrapped 95% confidence intervals for within-participants task differences (bootstrapped

at the participant level). *$p < .05$, **$p < .01$, ***$p < .001$, two-sided nonparametric randomization test.

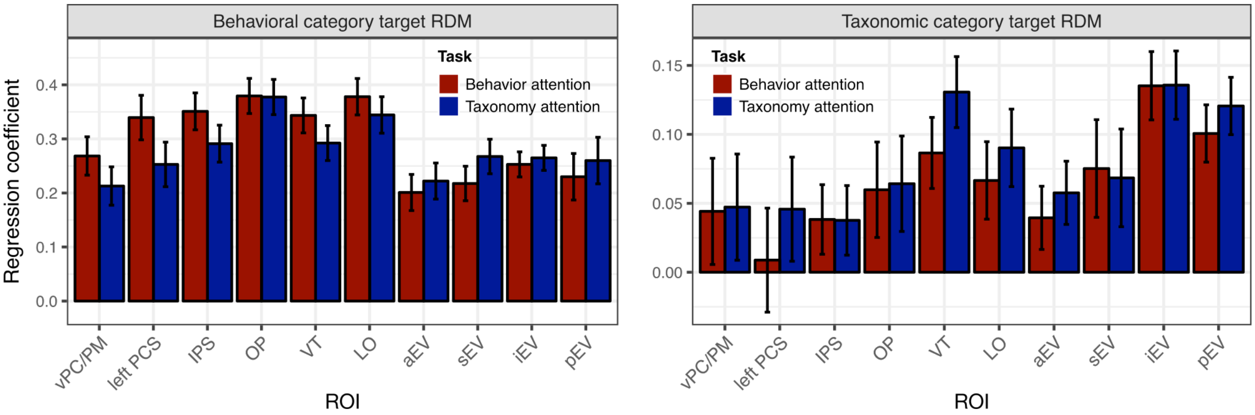

Supplementary Figure 8. Representational similarity analysis using standardized rank regression. Neural representational geometry in each ROI was modeled as a weighted sum of the behavioral category and taxonomic category target RDMs. Mean regression coefficients for the behavioral category target RDM and taxonomic category target RDM are plotted for both task condition. Error bars indicated bootstrapped 95% confidence intervals for within-participants task differences (bootstrapped at the participant level). This method is identical to the multiple regression used in the searchlight analysis (Figure 2), and reflects qualitatively similar results to the simpler approach using Spearman correlation reported in Figure 3.

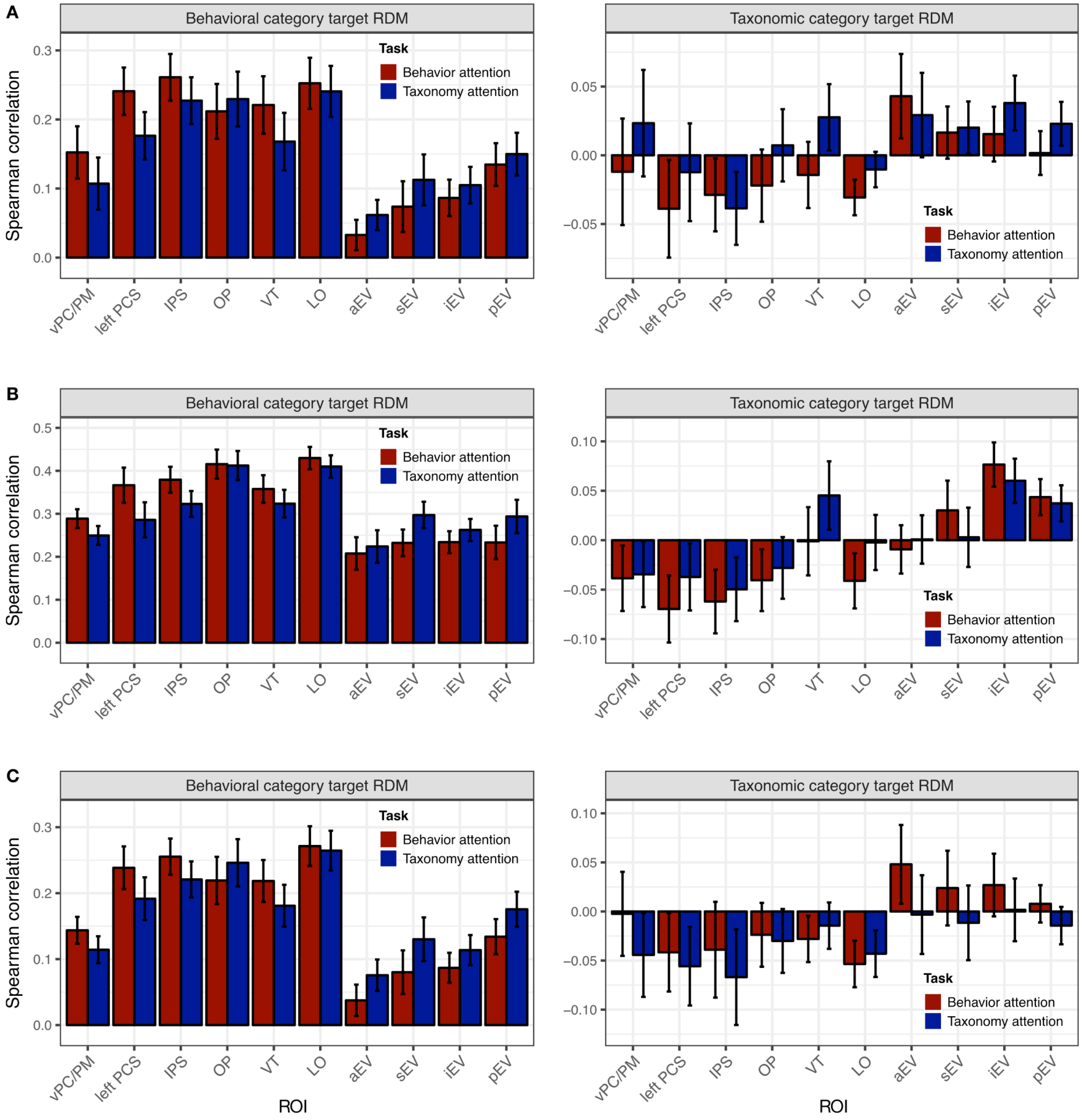

Supplementary Figure 9. Representational similarity analysis using alternative distance metrics and cross-validation schemes (cf. Figure 3). Neural RDMs for each ROI are compared to the categorical target RDMs using Spearman correlation. All error bars indicate bootstrapped 95% confidence intervals for within-participants task differences (bootstrapped at the participant level). (*A*) Neural RDMs were constructed using Euclidean distance to compute the pairwise dissimilarities between response patterns (rather than correlation

distance as in Figure 3). Response patterns were estimated for all five scanning runs for each attention task and neural RDMs were not computed in a cross-validation fashion (as in Figure 3). (*B*) Neural RDMs were constructed using leave-one-run-out cross-validation and correlation distance. Response patterns were estimated separately for each scanning run. For each cross-validation fold, response patterns for four runs were averaged, and pairwise correlation distances were computed between conditions in the averaged runs and the left-out fifth run (for each attention task). This results in a neural RDM with a nonzero diagonal. (*C*) Neural RDMs were constructed using the same leave-one-run-out cross-validation scheme, but using Euclidean distance as the pairwise dissimilarity metric.

Description	Behavioral category	Taxonomic category
Chimpanzee eating a fruit	Eating	Primate
Howler monkey eating leaves	Eating	Primate
Llama eating cactus fruits	Eating	Ungulate
Reindeer grazing on grass	Eating	Ungulate
Lammergeier eating carrion	Eating	Bird
Hummingbird drinking from flower	Eating	Bird
Chameleon eating grasshopper	Eating	Reptile
Komodo dragon eating carcass	Eating	Reptile
Caterpillar eating its own eggshell	Eating	Insect
Ladybug eating mites	Eating	Insect
Baboons fighting on rocks	Fighting	Primate
Geladas fighting amongst herd	Fighting	Primate
Bison butting heads on prairie	Fighting	Ungulate
Ibex locking horns on mountainside	Fighting	Ungulate
Seabirds fighting on rocks	Fighting	Bird
Vultures fighting in the snow	Fighting	Bird
Chameleon biting another chameleon	Fighting	Reptile
Komodo dragons fighting	Fighting	Reptile
Ant and ladybug fighting	Fighting	Insect
Stag beetles locking mandibles	Fighting	Insect
Baboon running toward water	Running	Primate
Monkey running away through tall grass	Running	Primate
Juvenile ibex running down mountainside	Running	Ungulate
Topi running through herd	Running	Ungulate
Penguin running across meadow	Running	Bird
Seagull running through cloud of insects	Running	Bird
Komodo dragon walking on rocks	Running	Reptile
Lizard running across sand	Running	Reptile
Ants traveling across sand	Running	Insect
Beatle running across dirt	Running	Insect
Macaque swimming underwater	Swimming	Primate
Snow monkey swimming in hot spring	Swimming	Primate
Deer swimming across lake	Swimming	Ungulate
Reindeer herd swimming across strait	Swimming	Ungulate
Duck swimming across stream	Swimming	Bird
Penguin swimming underwater	Swimming	Bird
Marine iguana swimming in clear water	Swimming	Reptile

Sea turtle swimming near seafloor	Swimming	Reptile
Dobsonfly larva swimming toward streambed	Swimming	Insect
Water beetle swimming underwater	Swimming	Insect

Supplementary Table 1. Descriptions of video clip stimuli and condition assignments. Each of the 40 video clip exemplars is briefly described. The condition assignments are indicated for each clip. There were two exemplar clips for each condition.

Parcel	Color	Extent	Task differences in Spearman's ρ (z-value)	
			Behavioral RDM	Taxonomic RDM
pEV	purple	1,419	-0.944	0.914
iEV	teal	1,321	-0.928	-0.191
sEV	olive	1,220	-2.142*	-0.798
aEV	red	882	-0.863	0.922
LO	gold	1,333	1.652	1.707
VT	maroon	2,063	2.326*	2.567*
OP	blue	3,570	0.372	0.223
IPS	copper	2,638	2.535*	0.770
Left PCS	green	1,356	2.784**	2.095*
vPC/PM	orange	3,995	2.228*	0.649
dPC	cyan	4,840	2.385*	2.221*
pSTS	white	2,793	1.135	0.365
Right dlPFC	light yellow	11,362	1.579	0.846
Left dlPFC	violet	5,199	1.856	0.739
mV	yellow	2,671	-0.211	0.434
Precuneus	brown	4,428	1.933	1.238
Cingulate	dark pink	3,166	1.731	0.909
OFC	navy	5,611	1.656	0.849
mPFC	dark gray	3,334	0.190	0.425

Supplementary Table 2. Task differences in Spearman correlation for all 19 parcels (Fig. 3). Parcels are listed roughly proceeding from posterior early visual areas anteriorly along the lateral surface, followed by medial structures. Parcel colors reference Supplementary Fig. 4*B*. Extent indicates the number of voxels referenced by all surface-based searchlights in the parcel. The average extent across all 19 parcels was 3,260 voxels (SD = 2,378 voxels). Note that neighboring searchlights overlap spatially and may overlap in the voxels they reference, although these voxels are only counted once for analysis purposes and in each parcel's extent. Task differences in representational geometry were evaluated by applying (exact) permutation tests to the Fisher transformed Spearman correlations between the observed neural RDM for each parcel and the behavioral category and taxonomic category target RDMs. Reported z-values were derived from the *p*-values returned by the nonparametric randomization test.

Negative values indicate decreased Spearman correlation with a target RDM when attending to the corresponding semantic information. Parcel label abbreviations are as follows. pEV: bilateral posterior early visual cortex comprising the occipital pole and posterior lateral occipital sulcus; iEV: bilateral inferior early visual cortex extending from the inferior bank of the posterior calcarine sulcus across the posterior lingual gyrus and posterior transverse collateral sulcus to the inferior occipital gyrus; sEV: bilateral superior early visual cortex encompassing the posterior calcarine sulcus and posterior cuneus; aEV: bilateral anterior early visual cortex including the anterior calcarine sulcus and a portion of the lingual gyrus; LO: bilateral lateral occipitotemporal cortex including the inferior middle occipital gyrus (and human MT+); VT: bilateral ventral temporal cortex including the fusiform gyrus, inferior temporal gyrus, and lateral occipitotemporal sulcus; OP: bilateral occipitoparietal and posterior parietal cortex extending from the lateral occipital sulcus dorsally to the transverse parietal sulcus; IPS: bilateral anterior intraparietal sulcus including the superior parietal lobule; left PCS: left postcentral sulcus, including the postcentral gyrus, inferior parietal lobule (supramarginal gyrus), and anterior intraparietal sulcus; vPC/PM: bilateral ventral pericentral gyri including the ventral central sulcus, premotor cortex, and extending ventrally to include the subcentral gyrus and posterior insula; dPC: bilateral dorsal pericentral gyri and central sulcus extending medially to the paracentral gyrus and posterior medial frontal gyrus; pSTS: bilateral posterior superior temporal sulcus including the posterior middle temporal gyrus and superior temporal gyrus; left dlPFC: left dorsolateral prefrontal cortex extending from the superior frontal gyrus ventrally to the inferior frontal gyrus and extending dorsomedially to the middle anterior medial superior frontal cortex; right dlPFC: right dorsolateral prefrontal cortex extending from the superior frontal gyrus ventrally to inferior frontal gyrus and extending dorsomedially to middle-anterior medial superior frontal cortex, as well as bilateral anterior superior temporal sulcus (aSTS) and middle temporal gyrus, and bilateral temporoparietal junction (TPJ), including the inferior

parietal lobule, supramarginal gyrus, and angular gyrus; mV: bilateral medial visual cortex extending from the parietooccipital sulcus across the anterior calcarine sulcus to the parahippocampal gyrus and medial aspect of the fusiform gyrus; Precuneus: bilateral precuneus including subparietal cortex and the marginal ramus of the cingulate sulcus, as well as the bilateral posterior superior frontal sulcus; Cingulate: bilateral middle cingulate cortex, medial subcortical structures, and the right anterior insula; OFC: bilateral orbitofrontal cortex extending posteriorly to include bilateral anterior temporal lobes (ATL; parahippocampal gyrus and temporal pole); mPFC: bilateral medial prefrontal cortex including the anterior cingulate and superior frontal gyrus. See Supplementary Table 3 for tests computed separately for each bilateral homologue and otherwise anatomically discontiguous parcel. *$p < .05$, **$p < .01$, two-sided nonparametric randomization test, uncorrected.

Parcel	Hemisphere	Color	Extent	Task differences in Spearman's ρ (z-value)	
				Behavioral RDM	Taxonomic RDM
pEV	L	purple	712	-1.309	-0.098
	R	purple	707	-0.827	0.596
iEV	L	teal	729	-0.233	0.681
	R	teal	552	-1.414	-0.777
sEV	L	olive	654	-2.235*	-1.162
	R	olive	566	-1.725	-0.991
aEV	L	red	470	0.191	1.917
	R	red	412	-1.339	-1.285
LO	L	gold	592	2.034*	1.411
	R	gold	741	0.061	0.771
VT	L	maroon	1,037	2.308*	2.038*
	R	maroon	1,026	1.929	2.001*
OP	L	blue	1,878	0.474	0.406
	R	blue	1,692	0.328	0.408
IPS	L	copper	980	0.378	0.130
	R	copper	1,658	2.602**	0.692
Left PCS	L	green	1,356	2.784**	2.095*
vPC/PM	L	orange	1,953	2.354*	1.135
	R	orange	2,042	1.454	0.125
dPC	L	cyan	2,616	2.074*	2.160*
	R	cyan	2,224	1.704	2.095*
pSTS	L	white	1,300	1.921	0.283
	R	white	1,493	0.435	0.553
Right dlPFC	R	light yellow	5,004	1.532	0.562
aSTS	L	light yellow	1,726	1.048	0.853
	R	light yellow	2,169	1.461	0.906
TPJ	L	light yellow	766	1.630	0.845
	R	light yellow	1,117	1.488	-0.113
Left OFC	L	light yellow	195	0.162	-0.101
Right aI	R	light yellow	138	1.691	-0.070
Left PreC	L	light yellow	127	1.962*	2.166*
Left dlPFC	L	violet	5,199	1.856	0.739
mV	L	yellow	1,374	-0.261	0.511
	R	yellow	1,297	0.112	0.494
Precuneus	L	brown	1,440	0.831	0.742
	R	brown	1,449	1.126	0.851

pSFS	L	brown	803	3.487***	1.546
	R	brown	653	0.465	0.062
Cingulate	L	pink	1,380	0.677	1.358
	R	pink	1,234	1.725	0.841
Left aI	L	pink	552	2.079*	-0.569
OFC	L	navy	2,275	1.393	0.905
	R	navy	2,083	1.550	0.578
mPFC	L	dark gray	1,649	0.507	0.579
	R	dark gray	1,685	0.077	0.287

Supplementary Table 3. Task differences in Spearman correlation computed separately for each anatomically discontiguous parcel. In many cases, the clustering algorithm returned bilateral homologues as one cluster, while in several cases additional spatially discontiguous regions of the cortical surface were included in a single cluster. We split these discontiguous regions into separate parcels based on the neighborhood structure of the cortical surface mesh, then analyzed each parcel separately using nonparametric randomization tests. The average extent across all discontiguous parcels was 1,394 voxels (SD = 1,026 voxels). Z-values were derived from the *p*-values returned by the randomization test, and negative values indicate decreased Spearman correlation with a target RDM when attending to the corresponding semantic categories. In addition to bilateral homologues, the highly diffuse right dlPFC cluster split into bilateral anterior superior temporal sulcus (aSTS) parcels, bilateral temporoparietal junction (TPJ) parcels, and three small parcels in left orbitofrontal cortex (OFC), right anterior insula (aI), and left precentral gyrus (PreC). The Precuneus cluster included bilateral posterior superior frontal sulcus (pSFS) parcels, and the Cingulate cluster included a portion of the left anterior insula (aI). *$p < .05$, **$p < .01$, ***$p < .005$, two-sided nonparametric randomization test, uncorrected.

Parcel	Task enhancement for within-category distances	
	Within-behavior	Within-taxon
pEV	-1.287	0.606
iEV	-0.664	0.145
sEV	-2.200*	-0.659
aEV	-0.696	0.534
LO	0.899	1.586
VT	2.200*	2.620**
OP	0.563	1.301
IPS	1.917	1.390
Left PCS	3.097***	2.584**
vPC/PM	2.705**	1.134
dPC	2.090*	2.620**
pSTS	0.632	0.382
Right dlPFC	1.770	1.113
Left dlPFC	1.617	1.023
mV	-0.452	0.669
Precuneus	1.873	1.542
Cingulate	1.501	1.278
OFC	1.669	1.207
mPFC	0.579	0.781

Supplementary Table 4. Task enhancement for within-category correlation distances for all 19 parcels (Fig. 4). Reported *z*-values were derived from the *p*-values returned by the nonparametric randomization test. Positive values in the “within-behavior” column can be interpreted as either decreased within-behavioral category distances when attending to behavior or an increase in between-taxonomic category distances when attending to taxonomy; similarly, positive values in the “within-taxonomy” column can be interpreted as either decreased within-taxonomic category distances when attending to taxonomy or increased between-behavioral category distances when attending to behavior. Negative values indicate the inverse effect. See Supplementary Table 5 for tests computed separately for each bilateral homologue and otherwise anatomically discontiguous parcel. $^{*}p < .05$, $^{**}p < .01$, $^{***}p < .005$, two-sided nonparametric randomization test, uncorrected.

Parcel	Hemisphere	Task enhancement for within-category distances	
		Within-behavior	Within-taxon
pEV	L	-1.601	-0.391
	R	-1.036	0.323
iEV	L	-0.094	0.376
	R	-0.794	-0.278
sEV	L	-2.221*	-0.985
	R	-2.019	-0.669
aEV	L	0.141	1.385
	R	-1.001	-0.994
LO	L	1.918	1.461
	R	-0.582	0.866
VT	L	2.364*	2.221*
	R	1.084	2.124*
OP	L	1.301	1.230
	R	-0.341	0.948
IPS	L	0.937	1.270
	R	1.891	0.241
Left PCS	L	3.097***	2.584**
vPC/PM	L	2.848**	1.626
	R	2.186*	0.377
dPC	L	1.941	2.640**
	R	1.600	2.015*
pSTS	L	1.499	0.470
	R	-0.024	0.407
Right dlPFC	R	1.623	0.908
aSTS	L	1.048	0.853
	R	1.461	0.906
TPJ	L	1.059	1.005
	R	1.396	1.431
Left OFC	L	0.292	-0.115
Right aI	R	1.856	-0.029
Left PreC	L	2.048*	2.243*
Left dlPFC	L	1.617	1.023
mV	L	-0.050	0.674
	R	0.254	0.314
Precuneus	L	0.836	0.855
	R	1.319	1.122

pSFS	L	3.182***	1.280
	R	0.997	0.446
Cingulate	L	0.602	1.430
	R	1.538	1.144
Left aI	L	1.895	-0.036
OFC	L	1.450	1.218
	R	1.546	1.033
mPFC	L	0.642	0.781
	R	0.493	0.728

Supplementary Table 5. Task enhancement for within-category distances computed separately for each anatomically discontiguous parcel. In addition to bilateral homologues, the highly diffuse right dlPFC cluster split into bilateral anterior superior temporal sulcus (aSTS) parcels, bilateral temporoparietal junction (TPJ) parcels, and three small parcels in left orbitofrontal cortex (OFC), right anterior insula (aI), and left precentral gyrus (PreC). The Precuneus cluster included bilateral posterior superior frontal sulcus (pSFS) parcels, and the Cingulate cluster included a portion of the left anterior insula (aI). *p < .05, **p < .01, ***p < .005, two-sided nonparametric randomization test, uncorrected.
